# Data Resource Profile: The Integrated Primary Care Information (IPCI) database, The Netherlands

**DOI:** 10.1093/ije/dyac026

**Published:** 2022-02-19

**Authors:** Maria A J de Ridder, Marcel de Wilde, Christina de Ben, Armando R Leyba, Bartholomeus M T Mosseveld, Katia M C Verhamme, Johan van der Lei, Peter R Rijnbeek

**Affiliations:** Department of Medical Informatics, Erasmus University Medical Center, Rotterdam, The Netherlands; Department of Medical Informatics, Erasmus University Medical Center, Rotterdam, The Netherlands; Department of Medical Informatics, Erasmus University Medical Center, Rotterdam, The Netherlands; Department of Medical Informatics, Erasmus University Medical Center, Rotterdam, The Netherlands; Department of Medical Informatics, Erasmus University Medical Center, Rotterdam, The Netherlands; Department of Medical Informatics, Erasmus University Medical Center, Rotterdam, The Netherlands; Department of Medical Informatics, Erasmus University Medical Center, Rotterdam, The Netherlands; Department of Medical Informatics, Erasmus University Medical Center, Rotterdam, The Netherlands

Key Features

- The Integrated Primary Care Information Project (IPCI) database contains longitudinal data of patients of a group of general practitioners (GPs) in The Netherlands.
- It consists of 2.5 million patient records, with median follow-up of 4.8 years, and includes patient demographics, information about contacts with GPs, symptoms, diagnoses, laboratory and clinical measurements, prescriptions and information on use of secondary care. The number of active patients at 1 July 2021 was 1 385 805.
- Started in 1992 by the Department of Medical Informatics of the Erasmus University Medical Center, Rotterdam, to enable better post-marketing surveillance of drugs, the IPCI database is used in a large number of studies including drug use, disease prevalence, drug safety and effectiveness studies and methodological research.
- The database is mapped to the Observational Medical Outcomes Partnership (OMOP) common data model to facilitate international collaboration using standardized analytics.
- Interested research collaborators can contact the IPCI project team at [www.ipci.nl].

## Data resource basics

### Dutch primary care data for research

Accessibility of health care is very good in The Netherlands. More than 99% of the population has health insurance^1^ and almost all citizens are registered with a general practitioner (GP). People are free to choose their GP. Enrolment at a general practice can be rejected because of capacity limitations or too great a distance between the practice and the patient’s address. The GP forms the point of care and acts as a gatekeeper to accessing secondary care. Over 12 months, around 78% of the population has at least one contact with their GP.^2^ Patient data in the GP records include demographic information, patient’s complaints and symptoms, diagnoses, laboratory test results, lifestyle factors and correspondence with secondary care, such as referral and discharge letters.

### The Integrated Primary Care Information database

**Figure 1:**
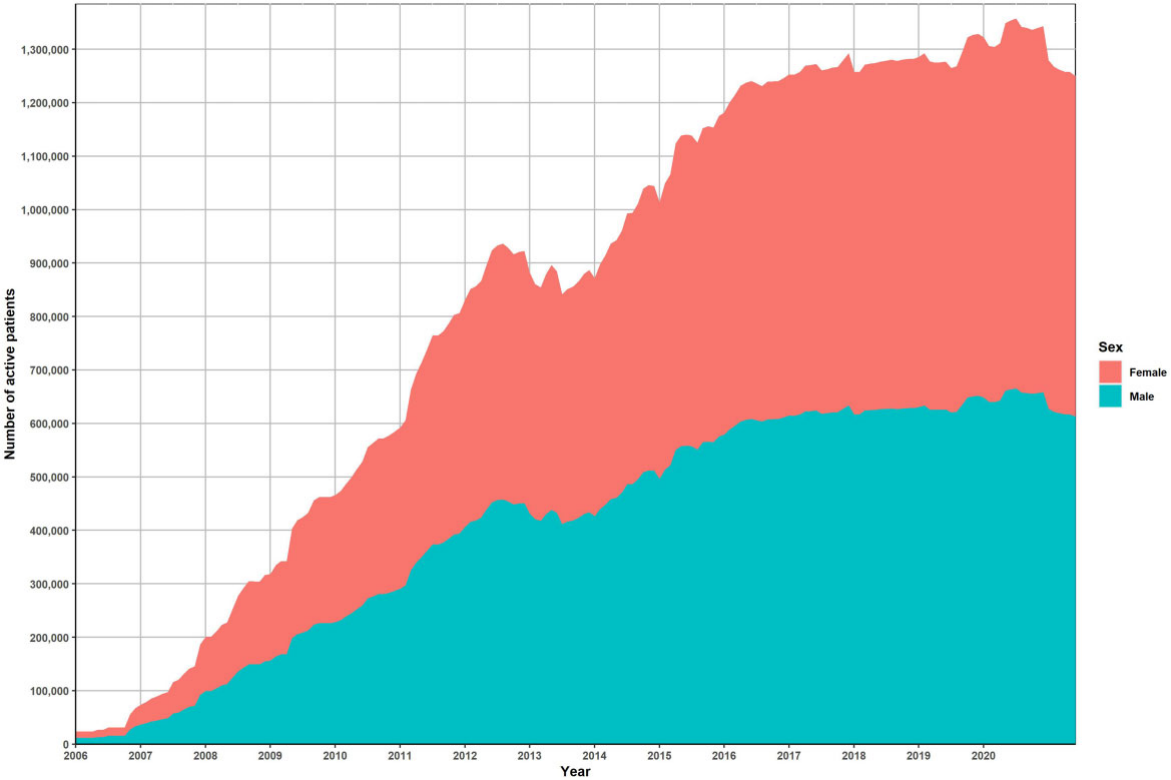
Size of the Integrated Primary Care Information Project (IPCI) database over calendar time

The Integrated Primary Care Information (IPCI) database is a database containing longitudinal, routinely collected data from computer-based patient records of around 350 GP practices throughout The Netherlands. The IPCI database was initiated in 1992 by the Department of Medical Informatics of the Erasmus University Medical Center in Rotterdam with the objective of enabling better post-marketing surveillance of drugs.^3^ In the first decade, the size of the database was limited. The current database includes patient records from 2006 onward, when the size of the database started to increase significantly (Figure 1). In 2016, IPCI was certified as a Regional Data Centre. Since 2019, the data have been standardized to the Observational Medical Outcomes Partnership common data model (OMOP CDM),^4^ enabling collaborative research in a large international network of databases using standardized analytics.

The primary goal of IPCI is to enable medical research. In addition, reports are generated to inform GPs and their organizations about the volume and type of provided care. Contributing GPs are encouraged to use this information for their internal quality evaluation.

The IPCI database is registered in the resources database of the European Network of Centres for Pharmacoepidemiology and Pharmacovigilance [ENCePP, coordinated by the European Medicines Agency (EMA)].^5^ Research is conducted according to the ENCePP Code of Conduct^6^ and the Guidelines on Good Pharmacovigilance Practices.^7^ If appropriate, research protocols are submitted to the European Union electronic register of Post-Authorisation Studies (EU-PAS) and the final study report is uploaded and available for consultation in the ENCePP registry.

### Data resource area and population coverage

GP practices included in the IPCI database are mainly located in the central part of the country, including the most densely populated area (the ‘Randstad’) but also non-urban areas. The IPCI database is a dynamic database in which patients are included from their registration at the GP practice until death or leaving the practice. In total, the database currently (1 July 2021) contains 2.5 million patients records with a median follow-up duration of 4.8 years. The number of active patients is 1.4 million, which comprises 8.1% of the Dutch population of 17 million (Table 1).

**Table 1:** Demographic characteristics of Integrated Primary Care Information Project (IPCI) patients, database release of 25 October 2021

	All patients	Active^a^ at 1 July 2021
Total	2 529 355	1 385 805
Sex		
Male	1 233 870 (48.8%)	679 877 (49.0%)
Female	1 295 485 (51.2%)	705 928 (51.0%)
Age		
<18		270 582 (19.5%)
18-64		839 742 (60.6%)
65+		275 481 (19.9%)
Urbanization^b^		
Extremely urbanized		324 581 (27.5%)
Strongly urbanized		273 156 (23.1%)
Moderately urbanized		245 016 (20.8%)
Hardly urbanized		232 618 (19.7%)
Not urbanized		104 728 (8.9%)
Urbanization unknown		205 706 (14.8%)
Follow-up, year, median (IQR)	4.8 (2.0–7.3)	6.3 (3.1–9.2)

a Alive and registered in the General Practitioner database.

b Because of privacy reasons, information about urbanization might be not completely up to date.

### Frequency of data collection

Every 6 months a new version of the IPCI database is released, but additional releases can be made if this is deemed necessary for certain research questions.

### Data governance, patient privacy

The IPCI database is under control of a governance board (named Raad van Toezicht IPCI database). Members of this board are GPs and external advisers. The Governance Board meets at least four times a year. The main task of the Board is to review each individual research proposal (see also under ‘Data Resource Access’). In addition, the Board is informed on all ongoing issues including the results of security scans, e.g. ISO (International Organization for Standardization) certification, and measures to comply with the Dutch General Data Protection Regulation (GDPR) such as the mandatory data protection impact analysis performed by the privacy office of Erasmus University Medical Center.

GPs are required to inform their patients about the use of their de-identified data for medical research. Patients can object to the use of their data and if this is the case, the data of these patients are not included in the research datasets.

IPCI informs all GPs about the studies being started. GPs may decide to withdraw the data of their practice for a specific study.

Data are collected, used and stored strictly in accordance with the GDPR.^8^ The Erasmus University Medical Center has people employed to control personal data protection and technical security. All medical data, including IPCI, are collected and stored according to the NEN7510 certificate, a Dutch standard for information security in health care.^9^

Almost all studies using IPCI data concern retrospective research of observational data, and as such are not subject to the Medical Research Involving Human Subjects Act (WMO) and do not require approval from a medical research ethical committee. If additional patient data have to be collected, the protocol is sent to an accredited medical research ethical committee for review. Before data are transferred to the central database, data are pseudonymized. The data are stored on an isolated network without internet connection. Within this network there are separate layers to distinguish access for data supply to GPs, data coding and data analysis. Data access is only possible at authorized computers in secured rooms and after authorized user access. Employees working with the IPCI and external researchers must sign a declaration of confidentiality. Only aggregated data are allowed to leave the secured environment.

### Funding sources

The IPCI project has no profit motive. To recover the cost of maintaining the database, projects are asked for a financial contribution. A data access fee is required to conduct research on the IPCI database. If IPCI staff are involved in the extraction, validation and analysis of IPCI data, additional fees might be needed.

In the period 2015–20, almost 40% of the studies using the IPCI data were investigator initiated without external financial support. Another 40% of the studies were supported by European funding programmes (e.g. Horizon 2020) and national funding programmes (e.g. ZonMw). Of the studies using IPCI data, 10% were Post Authorisation Safety Studies required by the European Medicines Agency. The remaining 10%of the studies were a mixture of studies sponsored by government (e.g. the Dutch Ministry of Health) or charitable organizations (e.g. Dutch Lung Foundation), and contract research for industry.

## Data collected

### Data source, transmission, conversion and anonymization

In The Netherlands, several information systems for GPs (Dutch: Huisarts Information System, HIS) are available. In the HIS, all the information needed in view of the patient’s care is collected (individual patient demographics, medical history, complaints and symptoms, diagnoses, laboratory results, lifestyle factors, referral notes to consultants, and hospital admissions). When a patient moves from one GP to another, all relevant data collected by the former GP are usually transferred. At each contact, the GP or practice staff will enter all relevant information into the patient’s file. These data consist of both coded information and free text. For complaints, symptoms and diagnoses, Dutch GPs use International Classification of Primary Care (ICPC-1) coding, an international standard developed and updated by the World Organization of Family Doctors’ (WONCA) International Classification Committee.^10^ In the HIS, correspondence from secondary care providers to the GP is also collected. Depending on the HIS GPs are using, this information is made available in the IPCI database partly as coded diagnoses and partly as textual information.

**Figure 2:**
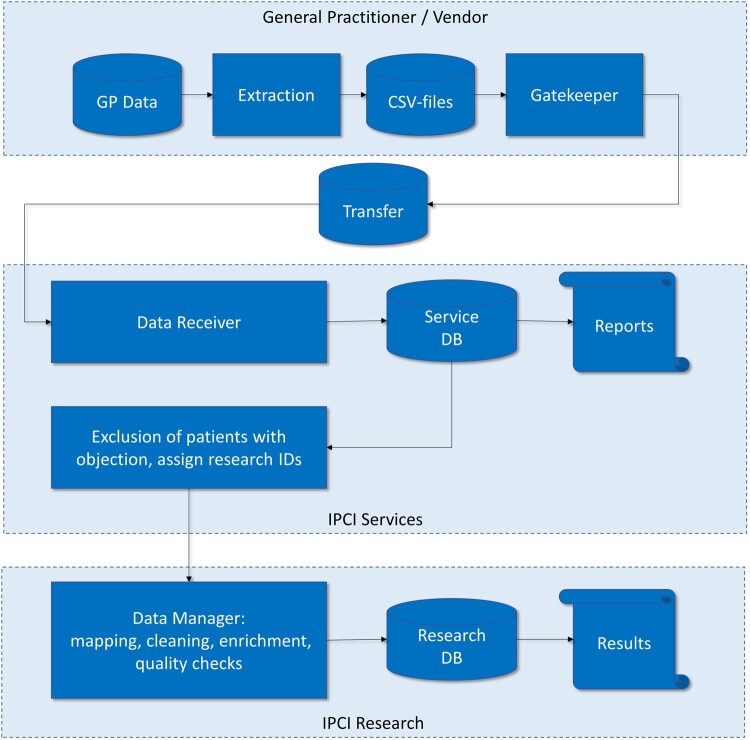
Data flow diagram. GP, general practitioner; CSV, comma-separated values; DB, database; ID, identification; IPCI, Integrated Primary Care Information Project

The pathway of transmission of data from the individual GP practice to the research database is shown in Figure 2. The intermediary called ‘IPCI Services’, separated from IPCI Research, ensures meticulous data protection. Before data transfer to IPCI Services, information in the electronic medical record that can be tied directly to individuals is removed, such as names, addresses and phone numbers, or is pseudonymized, such as dates of birth and death, which are rounded to months. If available, the postal code of the patient’s address is used to generate geospatial data such as ‘socially deprived area’ and ‘degree of urbanization’, and is then removed. Next, several data conversion and normalization steps are performed, to obtain a uniform dataset over all contributing HISs. After these steps, the resulting database is transferred to IPCI Research, structured as described in Table 2. Text is further processed to extract additional information, such as the date of hospitalization or discharge, and the specialty of the referral.

**Table 2:** Key elements of the Integrated Primary Care Information Project (IPCI) data tables

Data table	Information	Details
Patient	Patient type, sex, birth date, date of registration, date of and reason for leaving, date of death	For dates of birth and death, only month and year are provided
Contact	Type of contact, employee	
Diagnosis	Date, diagnosis code and free text	ICPC-1 coding
Measurement	Date, type of measurement, value of measurement This includes measurements directly taken at the GP office (e.g. height, weight, blood pressure, glucose level) as well as laboratory measurements, and lifestyle factors e.g. smoking habit and alcohol use	
Therapy	Date, therapy name, treatment code, dose, quantity, route, duration, indication	National product classification and WHO ATC coding
Communication	Referral and discharge letters	Not available for all practices as GP software system dependent. Mainly based on unstructured text
Actions	Date and type of medical procedures	
Unclassified	Free text notes entered by GP, other unstructured data.	

ICPC-1, International Classification of Primary Care; GP, general practitioner; WHO, World Health Organization; ATC, Anatomical Therapeutic Chemical (Classification System).

### Measures to ensure data quality

Prior to each data release, extensive quality control steps are performed, such as comparison of patient characteristics between practices and checks to identify abnormal temporal data patterns in practices. For each practice, around 200 quality indicators are obtained. Of these indicators, a quarter refer to population characteristics (e.g. reliability of birth and mortality rates). The other indicators are based on medical data (e.g. availability of durations of prescriptions, completeness of laboratory results, availability of hospital letters and prescriptions, proportion of patients with blood pressure measurement, etc). The indicators are combined using different weights depending on the importance of the item, in two quality scores for each practice—one for population information and one for medical content, both ranging between 1 and 10. Practices with one of these scores below 3 or the sum of the scores below 7 are excluded for research. The percentage of excluded practices usually is between 5–15%. This approach has shown to be very important, e.g. to check if data from practices that just joined the database are at an acceptable level of quality for epidemiological research.

### Standardization to the OMOP CDM

Like many other electronic health care record databases, IPCI has its own specific structure and uses one of several coding dictionaries (i.e. ICPC-1), which complicates collaborative research using other databases. To facilitate the conduct of multi-database research using standardized analytical tools, IPCI data are converted to the OMOP Common Data Model (OMOP CDM). This involves harmonization of the data structure and the terminology system standardized concepts while retaining the original source terminology. The OMOP CDM is developed and maintained by the Observational Health Data Sciences and Informatics (OHDSI) initiative and is described in detail on [https://ohdsi.github.io/CommonDataModel] and in The Book of OHDSI [http://book.ohdsi.org]. Vocabularies of the OMOP CDM are available on the OHDSI vocabularies repository Athena [https://athena.ohdsi.org/]. For the IPCI database, an Extraction, Transform, and Load (ETL) process has been developed following all the established best practices in the OHDSI community. This includes the use of the Data Quality Dashboard, an open-source R package [https://ohdsi.github.io/DataQualityDashboard] that reports potential quality issues in an OMOP CDM instance through the systematic execution and summarization of over 3300 configurable data quality checks.^11^ Interactive data profiling to assess patient demographics, the prevalence of conditions, drugs and procedures, and to evaluate the distribution of measurement values, can be done using the Achilles software [https://ohdsi.github.io/Achilles/].

## Data resource use

During the past 20 years, more than 200 research papers in peer-reviewed journals have been published using IPCI data. A full list of publications can be found on the IPCI website [www.ipci.nl]. Most research was performed with international collaborators in a multi-database setting. Many different research questions have been addressed, e.g. to describe patient populations and to assess safety and effectiveness of medicinal products. A considerable part to the work has focused on drug use (e.g. a study on prescriptions and adherence to asthma medication in children,^12^ a study reporting trends in prescribing antimicrobial drugs for urinary tract infections^13^ and a European study on use of antipsychotics in children and adolescents^14^). Other papers report incidence or prevalence of diseases such as Barrett’s oesophagus,^15^^,^^16^ lower urinary tract symptoms^17^ and, recently, a large international study on adverse events of special interest for COVID-19 vaccines.^18^ Several post-authorization safety studies are done in many therapeutic areas such as studies on non-steroidal anti-inflammatory drugs (NSAIDS).^19–22^ In addition, IPCI data are used for development of pharmaco-epidemiological methods.^23^^,^^24^

### International collaboration

Collaboration with other databases is seen as an important means to improve research, enable study of rare outcomes and replicate findings. Previously, IPCI participated in the EU-ADR project (Exploring and Understanding Adverse Drug Reactions by Integrative Mining of Clinical Records and Biomedical Knowledge), where researchers from academic institutions, policy authorities and the pharmaceutical industry worked together on several studies on drug safety.^25^ Using advanced information and communication technologies, electronic health records from databases in The Netherlands, Denmark, the UK and Italy were analysed according to common protocols and combined, while complying with privacy and data safety regulations. Based on this experience, a group of academic researchers formed the EU-ADR Alliance to perform post-authorization studies.^26–28^

The standardization to the OMOP CDM has further enabled international research at an unprecedented global scale due to the improved inter-operability of the data. The generation of reliable and reproducible evidence is facilitated by the powerful open-source analytical tools that are developed by the OHDSI community [https://ohdsi.org/software-tools/]. The OMOP CDM version of IPCI is used in multiple collaborative studies. For example, very recently the incidence of adverse events of special interest for COVID-19 vaccines across eight countries was characterized in a multi-national network cohort study,^18^ a prediction model on severe outcomes during COVID-19 infection was validated in 14 databases^29^ and IPCI participated in a large-scale characterization study of 4.5 million COVID-19 cases.^30^ The OMOP CDM version of IPCI is also used in several studies commissioned by the European Medicines Agency. Moreover, IPCI is participating in the European Health Data & Evidence Network EHDEN [https://www.ehden.eu/], a fast-growing network of data sources standardized to the OMOP CDM.

## Strengths and weaknesses

### Strengths

The IPCI database is a valuable source of real-world data from primary care, providing a wealth of information about drug use and disease prevalence, and allowing the conduct of association studies.

#### Size and follow-up

The IPCI database contains 2.5 million patient records which represent 8.1% of the Dutch population. This enables investigation of characteristics and associations which might be difficult to study in smaller datasets of clinical or population cohorts. The median follow-up duration is 4.8 years, interquartile range (IQR) 2.0 to 7.3, and this follow-up increases with each data release.

#### Representativeness

**Figure 3:**
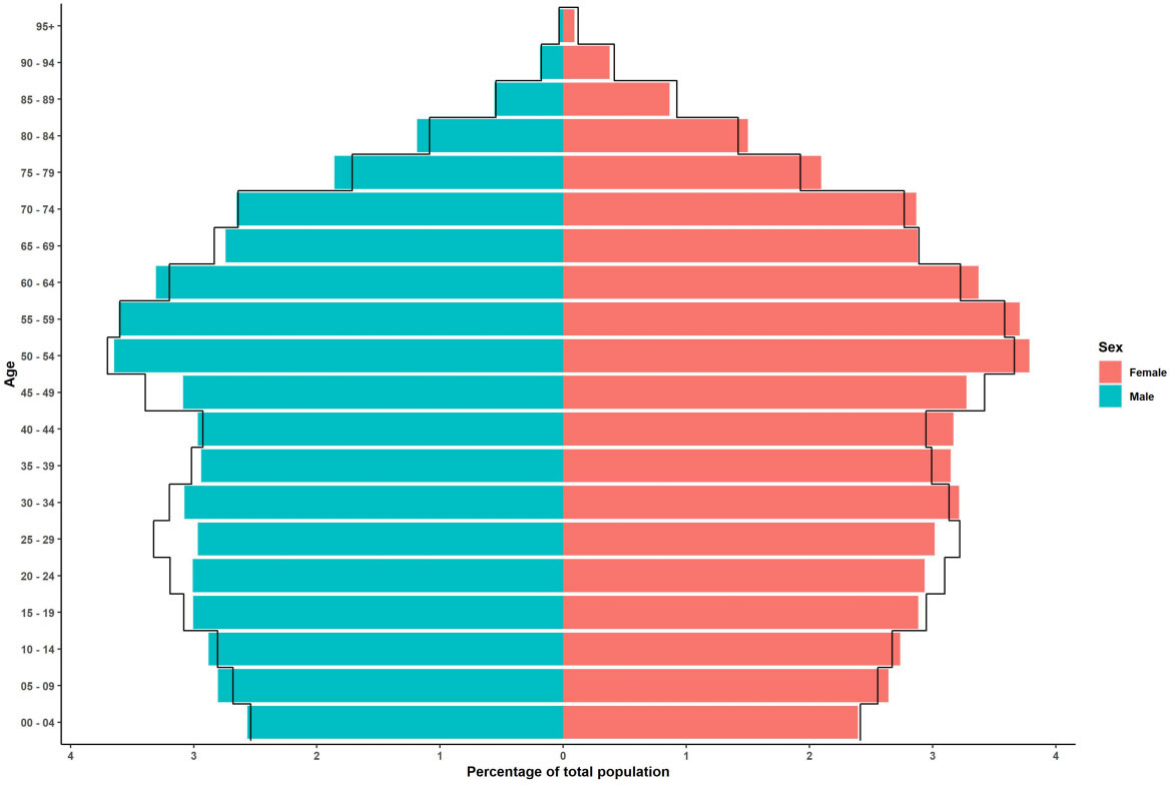
Population pyramid for the Integrated Primary Care Information Project (IPCI) in comparison with the Dutch population. Coloured bars: IPCI, active patients 1 July 2021. Black lines: Statistics Netherlands, 1 July 2020

The IPCI population is representative for the general Dutch population in terms of age and sex (see Figure 3). The geographical spread is limited, but GP practices are located both in urban and non-urban areas. There is no selection on type of health insurance or social economic status of patients.

#### Comprehensiveness

All patient information, as documented in the patient’s file at the GP practice, is available. Information from correspondence with secondary care and hospitals is available for about 25% of the practices, depending on the HIS used.

#### Textual information

The IPCI database does not only contain codes for symptoms and diagnoses, but also textual information entered by the GP. This can give more detailed information about the background, severity and certainty of the recorded diagnosis. Moreover, it enables validation of the coded information.

#### Data quality

For each database release, many checks are performed to monitor and benchmark the data of each GP practice. Practices have to meet quality conditions before their data can be included in the research dataset.

#### International collaboration, standardization to the OMOP CDM

The IPCI database has been used extensively in international collaborations demonstrating its value. These multi-database studies have been very useful to further improve the quality of the database and processes. The recent conversion to the OMOP CDM enables participation in larger international federated network studies within the OHDSI and EHDEN communities. The improved inter-operability of the data allows the use of standardized analytical pipelines, which improves transparency and reproducibility of the generated evidence and also reduces the time needed to conduct pharmaco-epidemiological research.

### Weaknesses

#### Missing data

The primary aim of data collection by GPs is patient management, and the data are therefore not customized for medical research purposes This implies that only information deemed to be relevant to the patient’s care is collected and entered into the patient’s file. Information about, for example, smoking habits, and height and weight measurements, is primarily collected in patients for whom knowledge on these variables is important to optimize patient care. The probability of data being missing will not be random but related to patient characteristics, consisting of both recorded and unrecorded information, making imputation of missing values difficult.

#### Information from secondary care

Information concerning secondary care or hospitalization is only available through correspondence with the GP. This implies that diagnoses, tests performed, length of hospital stays and medications prescribed in secondary care are not automatically available.

#### Data not captured

For reasons of patient confidentiality, information on race or ethnicity and geographical location is not available. For the same reason, exact dates of birth and mortality are not available, which may hamper certain analyses. Vaccinations administered outside the GP practice might be recorded by the GP, but completeness is not guaranteed. Vaccinations in the COVID-19 vaccination campaign are registered, but it is not yet clear to what extent.

#### Data validity

Because data in the GP systems are collected with patient management as the main goal, caution is needed when using IPCI data for research purposes. Diagnostic codes may be entered by the GP to indicate matters related to a previous diagnosis, or concerns of the patient. Some validation studies showed low positive predictive values for certain diagnostic codes.^28^^,^^31^^,^^32^ This means that in some studies, advanced disease algorithms or manual validation might be needed.

#### Heterogeneity

Because the data are collected in different GP information systems, there is heterogeneity in the available information. This involves, for example, information from secondary care, which can include correspondence, codes only or be completely lacking. Depending on the research question, it might be necessary to handle data from different GP information systems differently, or even restrict study data extraction to a selection of the systems.

#### Medication use

The data entered by the GPs concern prescription of medications. Information on drug dispensing is not available, nor is actual drug intake. If the duration of the prescription is not entered, it is calculated using the amount prescribed and the dosing information provided by the GP. Incorrectly calculated durations cannot be ruled out. The indication for the prescribed drug is not always available.

Medications prescribed in secondary care are not automatically available. Furthermore, data on use of over-the-counter drugs are not available. This should be taken into consideration when investigating drugs that may well be obtained outside the GP practice.

#### Data resource access

Researchers who want to use the IPCI data should contact the secretary [ipci@erasmusmc.nl] to obtain information about the procedure for gaining data access. A preliminary written request for research must be provided and this will be discussed with the database manager and a researcher from the IPCI team, focusing on design, feasibility and limitations. Next, a study proposal has to be generated using a pre-defined template, and submitted to the IPCI Governance Board for approval. The Board meets twice a year and will assess relevance, scientific quality and ethical aspects. Seeding trials will not be approved. Funding for the use of the data needs to be obtained by the researcher.

If the research protocol is approved, the researcher gains access to the IPCI data. The data are available in the original format of the IPCI research database or in the OMOP CDM format. Access is allowed only within the secured computer network after authorization through signing a declaration of confidentiality.

In the majority of multi-database studies, a federated approach is applied in which common analytical tools are run locally by the IPCI team and results are shared with the researcher. Only aggregated data can be exported from the secured environment. After the study is completed, data and scripts are archived at the department.

## Ethics approval

The IPCI database contains observational data, so research and reporting are not subject to the Medical Research Involving Human Subjects Act (WMO) and do not require approval from a medical research ethical committee.

## Data Availability

IPCI data are accessible under the conditions described in Data resource access.
